# Independent Expansion of Zincin Metalloproteinases in Onygenales Fungi May Be Associated with Their Pathogenicity

**DOI:** 10.1371/journal.pone.0090225

**Published:** 2014-02-28

**Authors:** Juan Li, Ke-Qin Zhang

**Affiliations:** Laboratory for Conservation and Utilization of Bio-resources, and Key Laboratory for Microbial Resources of the Ministry of Education, Yunnan University, Kunming, P.R. China; University of South Florida College of Medicine, United States of America

## Abstract

To get a comprehensive view of fungal M35 family (deuterolysin) and M36 family (fungalysin) genes, we conducted genome-wide investigations and phylogenetic analyses of genes in these two families from 50 sequenced Ascomycota fungi with different life styles. Large variations in the number of M35 family and M36 family genes were found among different fungal genomes, indicating that these two gene families have been highly dynamic through fungal evolution. Moreover, we found obvious expansions of Meps in two families of Onygenales: Onygenaceae and Arthodermataceae, whereas species in family Ajellomycetace did not show expansion of these genes. The strikingly different gene duplication and loss patterns in Onygenales may be associated with the different pathogenicity of these species. Interestingly, likelihood ratio tests (LRT) of both M35 family and M36 family genes suggested that several branches leading to the duplicated genes in dermatophytic and *Coccidioides* fungi had signatures of positive selection, indicating that the duplicated *Mep* genes have likely diverged functionally to play important roles during the evolution of pathogenicity of dermatophytic and *Coccidioides* fungi. The potentially positively selected residues discovered by our analysis may have contributed to the development of new physiological functions of the duplicated *Mep* genes in dermatophytic fungi and *Coccidioides* species. Our study adds to the current knowledge of the evolution of Meps in fungi and also establishes a theoretical foundation for future experimental investigations.

## Introduction

Many proteolytic proteases, such as extracellular serine proteases, aspartic proteases, and metalloproteases (Meps) are known to be important virulence factors related to fungal pathogenicity [1], [2]. Metalloproteinases, in which zinc is an essential metal ion for the catalytic activity, have been identified to play important roles in the pathogenicity of pathogenic fungi with different life styles, including vertebrate pathogens, entomopathogens and phytopathogens. For example, a 43.5-kDa Mep was identified from the dermatophytic fungus *Microsporum canis* and it could digest keratin azure during infection of cats and guinea pigs [3]. A thermolysin-like metalloproteinase produced by the entomopathogenic fungus *Metarhizium anisopliae* has been identified to not only cause insect cell and tissue damage [4], but also facilitate the development of entomopathogenic fungi within the infected insect host [5]. Moreover, a metalloprotease from the rice blast fungus *Magnaporthe grisea* was shown to function as a gene-for-gene-specific avirulence factor by directly binding the plant resistance gene product and triggering a signaling cascade leading to resistance [6]. These studies suggest that Meps have developed distinct functional properties to help fungi adapt to different ecological niches. Their functional diversities in different fungi raise interesting questions regarding the evolution of these important genes.

The Meps identified so far as secreted by pathogenic fungi mainly belong to two different families: the deuterolysin (M35) and the fungalysin (M36) [2]. These two families of proteases share little similarity with each other, with the exception of the HEXXH motif, in which two histidine residues function as the first and the second zinc ligand, respectively [7]. M35 family genes harbor a third zinc ligand known as an Asp that is found in a GTXDXXYG motif at the C-terminal, whereas M36 family genes utilize a Glu, which is present at the EXXXD motif at the C-terminal as the third zinc ligand [7], [8]. Previous evidence suggested that members of these two families are actual pathogenic factors in human infectious diseases. For example, *Aspergillus fumigatus*, an opportunistic human pathogen, can release a M36 family metalloprotease to help it invade mammalian lung [9]. In addition, at least ten *Mep* genes (designated as *Mep1* to *Mep10*) have been found in *Coccidioides posadasii*, which is a fungal respiratory pathogen of humans that can cause coccidioidomycosis (Valley fever) in immunocompromised individuals, and most of them were classified into M35 and M36 families. Moreover, the dermatophytic fungi, which cause various dermatophytosis common in animals and humans, can secrete several M36 family proteases with collagenolytic, elastinolytic and keratinolytic activities to help them survive in various niches [10]. Therefore, although the precise roles of these proteases from other phytopathogenic or entomopathogenic fungi have not yet been identified, we believed that the M35 and M36 family genes in different pathogenic fungi may have a wide variety of pathological actions. Thus, characterizing the evolution of M35 family and M36 family genes from different fungi can be of great importance for understanding the diversification of these *Mep* genes and for inferring their biological significance. Moreover, the increasing availability of completely sequenced fungal genomes also provides a good opportunity for us to characterize the *Meps* superfamily in different fungal taxa and to infer their evolutionary patterns.

Onygenales, a fungal order associated with a host/substrate shift from plants to animals, includes many important pathogenic fungi that can cause notorious diseases of vertebrates (e.g. *Coccidioides immitis*, *Coccidioides posadasii*, *Paracoccidioides brasiliensis*, *Histoplasma capsulatum*, *Trichophyton rubrum* and *Microsporum canis*) and also contain a number of non-pathogens (e.g. *Uncinocarpus reesii*) [11]. Previous studies suggested that both the M35 and M36 family genes have separately expanded in Onygenales [11], [12]. Our previous study also provided evidence that positive selection has acted on the duplicated M35 family genes in *Coccidioides* and promoted their novel functional adaptations (e.g., recent acquisition of virulence traits) of these species [13]. Phylogenetic analyses in this paper suggested that M35 and M36 family genes have undergone intriguing evolution in Onygenales fungi. To better understand the evolutionary force that have likely shaped the diversification of these duplicated M35 and M36 family genes in Onygenales species and the implications of such changes, we also investigated the possible selection pressures responsible for the duplicated genes in Onygenales species.

## Data and Methods

### Data sets

In order to understand the evolutionary history and functional divergence of the M35 and M36 family genes in fungi, we performed a comprehensive evolutionary analysis of these two family genes from the sequenced genomes of 50 Ascomycota fungi. These fungi have different life styles and they include saprophytes, opportunistic human pathogens, plant pathogens and entomopathogens. The genome sequences and their corresponding annotated proteins database of the 50 Ascomycota fungi are available from the Fungal Genome Initiative at the BROAD Institute (http://www.broad.mit.edu/annotation/fungi/fgi) or Fungal Genome Research (http://fungalgenomes.org/).

To avoid search bias, we applied two methods to find putative homologs of M35 family sequences and M36 family sequences from the fungal genome databases employed in this study. In the first method, the M35 family genes (*Mep2* to *Mep8*) and M36 family genes (*Mep9* and *Mep10*) from *C. posadasii* were used as query sequences to do TBLASTN and BLASTP searches with E-value cutoff of 10^−10^ against the genome nucleotide database and fungal protein database, respectively. The sequences that met the criteria of both searching procedures and with amino acid sequence identities greater than 30% and covering more than 150 amino acids were considered as candidate genes. In the second method, the HMM profile Peptidase_M35 (PF02102; http://pfam.sanger.ac.uk/family/PF02102#tabview=tab6) and Peptidase_M36 (PF02128; http://pfam.sanger.ac.uk/family/PF02128#tabview=tab6) were downloaded and used as queries for homologous protein searches using the program HMMSEARCH from the HMMER package (http://hmmer.wustl.edu/). For each search result, hits were considered significant when they matched the Pfam HMM profile with E values <10^−5^. In total, 105 M35 family genes and 58 M36 family genes were identified, including two M35 family genes and four M36 family genes from the Basidiomycete fungus *Coprinopsis cinerea* which were used as outgroups for these two families, respectively. The taxonomic information and the identified gene number of the fungal species are presented in Table 1.

**Table 1:** Number of both M35 family and M36 family genes in different fungal species.

Species	Host	Taxonomy	M35 family	M36 family	Total
*Coccidioides immitis*	vertebrate pathogen	Eurotiomycetes:Onygenales:Onygenaceae	7	2	9
*Coccidioides posadasii*	vertebrate pathogen	Eurotiomycetes:Onygenales:Onygenaceae	7	2	9
*Uncinocarpus reesii*	saprophyte	Eurotiomycetes:Onygenales:Onygenaceae	4	2	6
*Microsporum canis*	vertebrate pathogen	Eurotiomycetes:Onygenales:Arthodermataceae	5	5	10
*Microsporum gypseum*	vertebrate pathogen	Eurotiomycetes:Onygenales:Arthodermataceae	5	5	10
*Trichophyton equinum*	vertebrate pathogen	Eurotiomycetes:Onygenales:Arthodermataceae	5	4	9
*Trichophyton rubrum*	vertebrate pathogen	Eurotiomycetes:Onygenales:Arthodermataceae	5	4	9
*Trichophyton tonsurans*	vertebrate pathogen	Eurotiomycetes:Onygenales:Arthodermataceae	5	5	10
*Paracoccidioides brasiliensis*	vertebrate pathogen	Eurotiomycetes:Onygenales:Ajellomycetaceae	1	-	1
*Histoplasma capsulatum*	vertebrate pathogen	Eurotiomycetes:Onygenales:Ajellomycetaceae	1	-	1
*Blastomyces dermatitidis*	vertebrate pathogen	Eurotiomycetes:Onygenales:Ajellomycetaceae	2	-	2
*Aspergillus flavus*	vertebrate pathogen	Eurotiomycetes:Eurotiales:Trichocomaceae	6	2	8
*Aspergillus fumigates*	vertebrate pathogen	Eurotiomycetes:Eurotiales:Trichocomaceae	3	1	4
*Neosartorya fischeri*	vertebrate pathogen	Eurotiomycetes:Eurotiales:Trichocomaceae	2	1	3
*Aspergillus terreus*	vertebrate pathogen	Eurotiomycetes:Eurotiales:Trichocomaceae	2	1	3
*Aspergillus clavatus*	saprophyte	Eurotiomycetes:Eurotiales:Trichocomaceae	1	1	2
*Aspergillus niger*	saprophyte	Eurotiomycetes:Eurotiales:Trichocomaceae	-	1	1
*Aspergillus nidulans*	saprophyte	Eurotiomycetes:Eurotiales:Trichocomaceae	4	-	4
*Aspergillus oryzae*	saprophyte	Eurotiomycetes:Eurotiales:Trichocomaceae	5	2	7
*Penicillium chrysogenum*	saprophyte	Eurotiomycetes:Eurotiales:Trichocomaceae	1	-	1
*Chaetomium globosum*	endophyte	Sordariomycetes	-	-	-
*Epichloë festucae*	endophyte	Sordariomycetes	-	-	-
*Fusarium graminearum*	phytopathogen	Sordariomycetes	1	1	2
*Fusarium oxysporum lycopersici*	phytopathogen	Sordariomycetes	2	1	3
*Fusarium solani*	phytopathogen	Sordariomycetes	2	1	3
*Fusarium verticillioides*	phytopathogen	Sordariomycetes	2	1	3
*Magnaporthe grisea*	phytopathogen	Sordariomycetes	3	2	5
*Metarhizium anisopliae*	entomopathogen	Sordariomycetes	1	-	1
*Neurospora crassa*	saprophyte	Sordariomycetes	2	-	2
*Podospora anserine*	saprophyte	Sordariomycetes	-	-	-
*Verticillium albo-atrum*	phytopathogen	Sordariomycetes	4	2	6
*Verticillium dahlia*	phytopathogen	Sordariomycetes	3	2	5
*Trichoderma reesei*	saprophyte	Sordariomycetes	1	1	2
*Trichoderma atroviride*	saprophyte	Sordariomycetes	1	1	2
*Trichoderma virens*	saprophyte	Sordariomycetes	1	1	2
*Phaeosphaeria nodorum*	phytopathogen	Dothideomycetes	2	2	4
*Pyrenophora tritici-repentis*	phytopathogen	Dothideomycetes	1	1	2
*Botryotinia fuckeliana*	phytopathogen	Leotiomycetes	2	-	2
*Botrytis cinerea*	phytopathogen	Leotiomycetes	2	-	2
*Sclerotinia sclerotiorum*	phytopathogen	Leotiomycetes	2	-	2
*Candida albicans*	vertebrate pathogen	Saccharomycotina	-	-	-
*Candida glabrata*	vertebrate pathogen	Saccharomycotina	-	-	-
*Candida tropicalis*	vertebrate pathogen	Saccharomycotina	-	-	-
*Debaryomyces hansenii*	saprophyte	Saccharomycotina	-	-	-
*Pichia stipitis*	saprophyte	Saccharomycotina	-	-	-
*Saccharomyces cerevisiae*	saprophyte	Saccharomycotina	-	-	-
*Yarrowia lipolytica*	saprophyte	Saccharomycotina	-	-	-
*Schizosaccharomyces japonicus*	saprophyte	Taphrinomycotina	-	-	-
*Schizosaccharomyces pombe*	saprophyte	Taphrinomycotina	-	-	-
*Coprinopsis cinerea*	saprophyte	Basidiomycota: Agaricomycotina	2	4	6

### Phylogenetic analysis

For each of the two families of genes, MUSCLE v3.5 was used to generate protein alignment with default settings [14]. The ambiguous areas of alignments were located and removed by using the program Gblocks 0.91b [15], [16] with default parameters. The gap selection criterion “with half” was used here.

Phylogenetic analyses of the alignments were performed using PHYML 3.0 [17] for Maximum likelihood (ML) analysis and MrBayes 3.1.2 [18] for Bayesian inference. In the ML analysis, the best-fitting model of sequence evolution were estimated for both M35 family and M36 family genes using program ProtTest [19]. The chosen model WAG+I+G and their parameters (I = 0.03, G = 1.912 for M35 family genes; I = 0.038, G = 1.149 for M36 family genes) were used in the ML analysis. The reliability of the tree topology was evaluated using bootstrap support [20] with 100 for ML analysis. In addition, Bayesian inference was conducted using MrBayes 3.1.2 [18]. The best-fitting model of sequence evolution determined by ProtTest [19] was also used as the priors of Bayesian inference. Bayesian analysis started with randomly generated trees and Metropolis-coupled Markov chain Monte Carlo (MCMC) analyses were run for 2×10^6^ generations. The run was stopped when the average standard deviation of split frequencies was less than 0.01 in all the cases (MrBayes 3.1.2 manual). To ensure that these analyses were not trapped in local optima, the dataset was run three times independently. We determined the burn-in period by checking for likelihood stability. A 50% majority rule consensus of post burn-in trees was constructed to summarize posterior probabilities (PPs) for each branch.

### Gene duplication and loss analyses

To investigate the gene duplication and loss scenarios of M35 and M36 family genes in Onygenales, we used Notung 2.6 [21], [22] to reconcile the species tree and gene tree. The species tree was inferred based on a combined alignment of six genes from each Onygenales species as those used in James et al [23], including 18S rRNA, 28S rRNA, ITS RNA, translation elongation factor 1-α (TEF1α), RNA polymerase II largest subunit (RPB1) and RNA polymerase II second largest subunit (RPB2) (9601-bp in total, data not shown). Phylogenetic analyses of the alignments were performed as described by Li et al [13] and the model TrN+I+G of sequence evolution was optimized using Akaike information criterion [24], [25] as implemented in Modeltest version 3.7 [26]. For the gene tree used here, we collapsed those inconsistent nodes produced by different tree-building methods, which were also poorly supported, into polytomies [27].

In addition, we investigated the gene duplication and loss scenarios of M35 genes in Eurotiales fungi. The species tree and gene tree of Eurotiales fungi were inferred by using the same methods mentioned above.

### Selective pressure analyses

**Figure 1:**
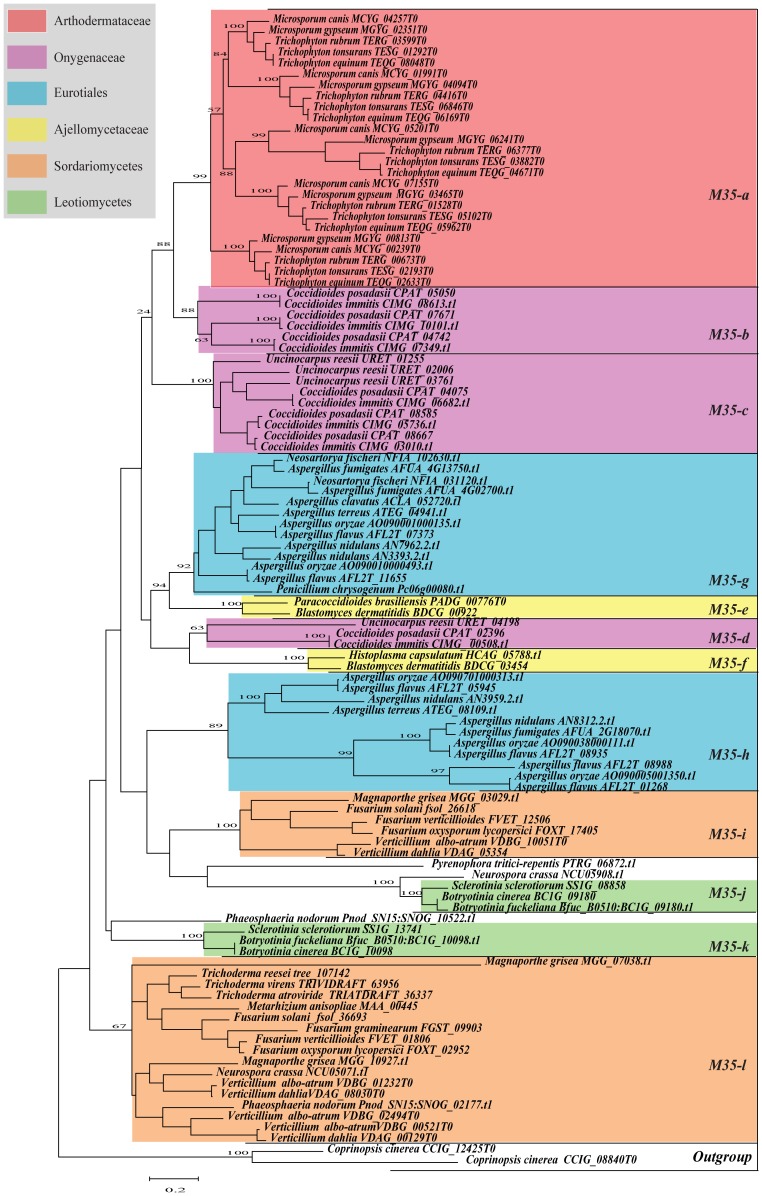
ML tree based on amino acid sequences of 105 M35 family genes. The tree was performed using PHYML 3.0[17]. The best-fitting model WAG+I+G and their parameters (I = 0.03, G = 1.912) which were estimated by program ProtTest [50] were used in the ML analysis. The reliability of the tree topology was evaluated using bootstrap support [20] with 100.

To investigate the possible selective forces behind M35 and M36 family gene duplications in Onygenales, we conducted likelihood ratio tests (LRT) for these two families respectively. From the phylogeny shown in Figure 1, we can see that multiple gene duplications of the M35 family genes have occurred in Eurotiales, and some of the duplication events occurred after the divergence of Eurotiales and Onygenales. To avoid the interference of within-Eurotiales duplications on our analyses, we used more distantly related Sordariomycetes fungi as outgroup to conduct LRTs analysis of M35 family genes. For M36 family genes, we still used Eurotiales fungi as outgroup. Finally, a 861-bp codon-based alignment for 62 M35 family genes (Fig. S1) and a 1569-bp codon-based alignment for 38 M36 family genes (Fig. S2) were used to construct phylogenetic trees using the same methods mentioned above except that the best-fitting model of sequence evolution used for ML and Bayesian analyses were estimated using program Modeltest 3.06 [26]. Because the likelihood analysis might be sensitive to tree topology used, we collapsed the nodes that showed inconsistent branching patterns from different tree-building methods and with poor statistical support into polytomies [27].

The ratio ω (dN/dS) is the ratio of the number of non-synonymous substitutions per non-synonymous site (dN) to the number of synonymous substitutions per synonymous site (dS), which provides an indication of the change in selective pressures [28]. A dN/dS ratio  = 1, <1, and >1 are indicative of neutral evolution, purifying selection, and positive selection on the protein involved, respectively [29], [30]. The codon substitution models implemented in the CODEML program in the PAML 4.4b package [31] were used to analyze changes of selective pressure. All models were corrected for transition/transversion rate and codon usage biases (F3×4). The ambiguous sites were removed in PAML analysis (clean data  =  yes). Different starting ω values were also used to avoid the local optima on the likelihood surface [32].

Two branch-specific models, i.e., “one-ratio” (M0) and “free-ratios”, were compared. The M0 model assumes the same ω ratio for all branches while the “free-ratios” model assumes an independent ω ratio for each branch [33]. We constructed LRT to compare the two models. Significant differences between models were evaluated by calculating twice the log-likelihood difference following a χ^2^ distribution, with the number of degrees of freedom equal to the difference in the numbers of free parameters between models.

Site-specific models, which allow for variable selection patterns among amino acid sites, M1a, M2a, M7, and M8, were used to test for the presence of sites under positive selection. The M2a and M8 models identify positively selected sites. When these 2 positive-selection models fitted the data significantly better than the corresponding null models (M1a and M7), the presence of sites with ω>1 would be suggested. The conservative Empirical Bayes approach [34] was then used to calculate the posterior probabilities of a specific codon site and identify those most likely to be under positive selection.

Considering that positive selection may act in very short episodes during the evolution of a protein [35] and affect only a few sites along a few lineages in the phylogeny, the likelihood models accommodating ω ratios to vary both among lineages of interest and among amino acid sites, as an improved version of the “branch-site” model [36], were also considered here. We used branch-site Model A as a stringency test (test 2) and identified amino acid sites under positive selection by an empirical Bayes approach along the lineages of interest [36], [37]. The log-likelihoods for the null and alternative models were used to calculate a likelihood ratio test statistic, which was then compared against the χ2 distribution (with a critical value of 3.84 at a 5% significance level) [31]. We used the conservative Bayes Empirical Bayes (BEB) approach, which assigns a prior to the model parameters and integrates over their uncertainties, to calculate the posterior probabilities of a specific codon site and to identify those most likely to be under positive selection. In addition, the Bonferroni correction [38], [39] was also applied for correcting multiple testing in the analysis according to the number of tests of significance performed.

## Results

### Taxonomic distribution of M35 and M36 gene families

As shown in Table 1, large variations of the gene numbers for both M35 and M36 family genes were observed from the analyzed Ascomycota species, suggesting these two gene families have been highly dynamic through fungal evolution.

For the M35 family, a total of 103 genes were identified from the analyzed Ascomycota fungal species. In Family Onygenaceae of the Onygenales Order, 7 genes were identified from the human pathogenic fungi *C. posadasii* and *C. immitis*, and 4 genes were predicted from the saprophytic fungus *U. reesii*. In Family Arthodermataceae of Onygenales, five genes each were identified from the five dermatophytic fungi (*M. canis*, *Microsporum gypseum*, *Trichophyton equinum*, *T. rubrum* and *Trichophyton tonsurans*). These fungi can cause dermatosis in humans and animals. In Family Ajellomycetace of Onygenales, only one M35 family gene each was predicted from *H. capsulatum* and *P. brasiliensis*, and two genes were identified from *Blastomyces dermatitidis*. This result suggested that M35 family genes have expanded in two families of Onygenales: Onygenaceae and Arthodermataceae. The genus *Aspergillus*, which includes species with very different life styles with some very harmful and others beneficial to humans, showed the most variations of gene numbers (range from 0 to 6). For other fungal species, most contained 1 to 4 M35 family genes. However, no M35 family gene was identified from several fungal groups (*e.g.* Saccharomycotina fungi, Taphrinomycotina fungi, two endophytic fungi and some saprophytic fungi) (Table 1).

For the M36 family, a total of 54 genes were identified from the analyzed Ascomycota fungal genome sequences. In Onygenales fungi, the dermatophytic fungi (Arthodermataceae) contained the most with 4–5 genes per species, while 2 genes each were identified from Onygenaceae fungi (*C. posadasii*, *C. immitis* and *U. reesii*) and no M36 family gene was identified from Arthodermataceae fungi *(H. capsulatum*, *P. brasiliensis* and *B. dermatitidis*), suggesting that M36 family genes might have expanded in dermatophytic fungi. For other fungal species, 1 or 2 M36 family genes were predicated. However, several fungal groups (including Saccharomycotina fungi, Taphrinomycotina fungi, two endophytic fungi, three Leotiomycetes fungi, *M. anisopliae*, *Neurospora crassa* and *Podospora anserine*) contain no obvious M36 family gene homologs (Table 1). These results suggest that the M36 family genes may have undergone many gene duplication and gene loss events during the evolution of Ascomycota fungi.

### Phylogenetic analysis of M35 family

Phylogenetic relationships among the M35 family genes were analyzed based on an alignment consisting of 185 amino acids from 105 M35 family genes (include 103 genes from Ascomycota fungi and 2 outgroup genes from the Basidiomycota fungus *C. cinerea*; Fig. S3) using maximum-likelihood and Bayesian techniques. As seen in Fig. 1 and Fig. S4, phylogenetic analyses consistently grouped the M35 family genes into 12 distinct clades, which are abbreviated as *M35-a* to *M35-l*. Among these clades, *M35-a* (BS = 99%; PP = 100%) is a dermatophyte-specific lineage, containing exclusively the M35 genes from five dermatophytic fungi with *Microsporum* spp. branching basally to *Trichophyton* spp. It seemed that the multiplication of these genes occurred before the divergence of these five species. This is because each subclade within this clade contained one gene from each species, suggesting that these genes had a conserved and essential function in dermatophytes. The genes from Onygenaceae fungi fell into three clades (*M35-b*, *M35-c* and *M35-d*). Clades *M35-c* (BS and PP = 100%) and *M35-d* (BS and PP = 100%) were comprised of sequences from *C. posadasii*, *C. immitis* and *U. reesii*, while *M35-b* (BS = 88%; PP = 98%) was a *Coccidioides*-specific lineage, consisted of three duplicated genes from each of the two *Coccidioides* species. The genes from Ajellomycetaceae fell into clades *M35-e* (BS and PP = 100%) and *M35-f* (BS and PP = 100%). Clade *M35-f*, which contained genes from *B. dermatitidis* and *P. brasiliensis*, was closely related to Onygenaceae lineage *M35-d*. In contrast, clade *M35-e*, which included one gene from *H. capsulatum* and another from *B. dermatitidis*, showed close relationships with Eurotiales lineage *M35-g* (BS = 92%; PP = 100%).

From our phylogenetic analysis, we found that except for Arthodermataceae and Onygenaceae, the M35 family genes from other Ascomycota fungi within the same categories were consistently clustered into two clades respectively. For example, the genes from Ajellomycetaceae fell into *M35-e* and *M35-f* clades and those from Eurotiales were clustered into *M35-g* and *M35-h* clades (BS = 89%; PP = 86%). Clades *M35-i* (BS and PP = 100%) and *M35-l* (BS = 67%; PP = 70%) were comprised of genes from Sordariomycetes while clades *M35-j* (BS and PP = 100%) and *M3-k* (BS and PP = 100%) mainly consisted of those from Leotiomycetes fungi. Noticeably, one gene from *Neurospora crassa* showed a close relationship with clade *M35-j*. Interestingly, although the genes from the same taxonomic groups of fungi were consistently clustered into two clades, gene duplication and loss events might have also occurred during their evolution, leading to the diversification of M35 family genes in different fungi. For instance, though clades *M35-i* and *M35-l* both contained genes from Sordariomycetes, clade *M35-l* contained more genes than clade *M35-i*.

### Phylogenetic analysis of M36 family

**Figure 2:**
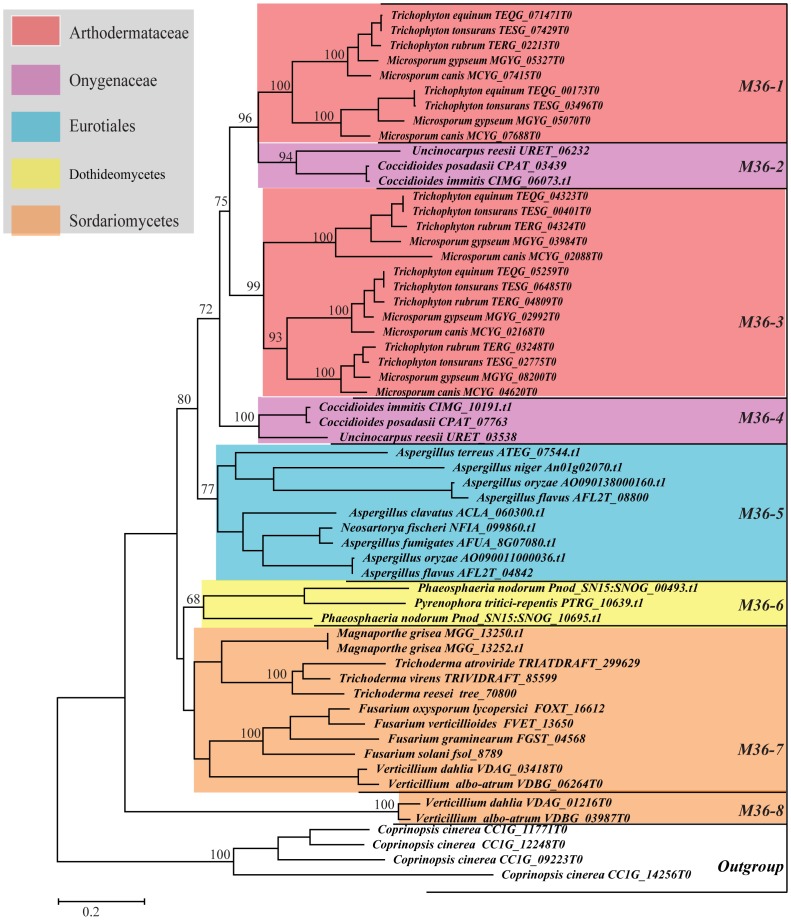
ML tree based on amino acid sequences of 58 M36 family genes. The tree was performed using PHYML 3.0[17]. The best-fitting model WAG+I+G and their parameters (I = 0.038, G = 1.149) which were estimated by program ProtTest [50] were used in the ML analysis. The reliability of the tree topology was evaluated using bootstrap support [20] with 100.

Results from our phylogenetic analyses based on an alignment consisting of 538 amino acids from 58 M36 family sequences (include 54 genes from Ascomycota fungi and 4 outgroup genes from the Basidiomycota fungus *C. cinerea*, Fig. S5) were largely consistent with the taxonomical relationships among the major fungal groups. The results suggested that the Dothideomycetes showed close relationships with Sordariomycetes and that the Eurotiales species were closely related to Onygenales fungi. Phylogenetic analyses based on the M36 family genes consistently showed eight strongly-supported monophyletic clades which were designated as *M36-1* to *M36-8* in the trees (see Figure 2 and Fig. S6). The M36 family genes from Arthodermataceae fungi (*M. canis*, *M. gypseum*, *T. equinum*, *T. rubrum* and *T. tonsurans*) and Onygenaceae fungi fell into two clades, respectively. Among these clades, clades *M36-1* (BS and PP = 100%) and *M36-3* (BS = 99%; PP = 100%) each contained the M36 genes from dermatophytic fungi. These genes seemed to have duplicated before the divergence of these five dermatophytic species. The sequences from Onygenaceae fungi (*C. posadasii*, *C. immitis* and *U. reesii*) grouped into clades *M36-2* (BS = 94%; PP = 100%) and *M36-4* (BS and PP = 100%), and their duplication likely occurred after the divergence of Onygenaceae and Arthodermataceae families. The genes from Eurotiales and Dothideomycetes fell into clades *M36-5* (BS = 77%; PP = 94%) and *M36-6* (BS = 68%; PP = 99%), respectively. The genes from Sordariomycetes were mainly grouped into clades *M36-7* and *M36-8* (BS and PP = 100%). However, clade *M36-8* was comprised of the duplicated genes from *Verticillium albo-atrum* and *Verticillium dahlia*, showing distant divergence with other clades.

### Gene duplication and loss analysis in Onygenales and Eurotiales

**Figure 3:**
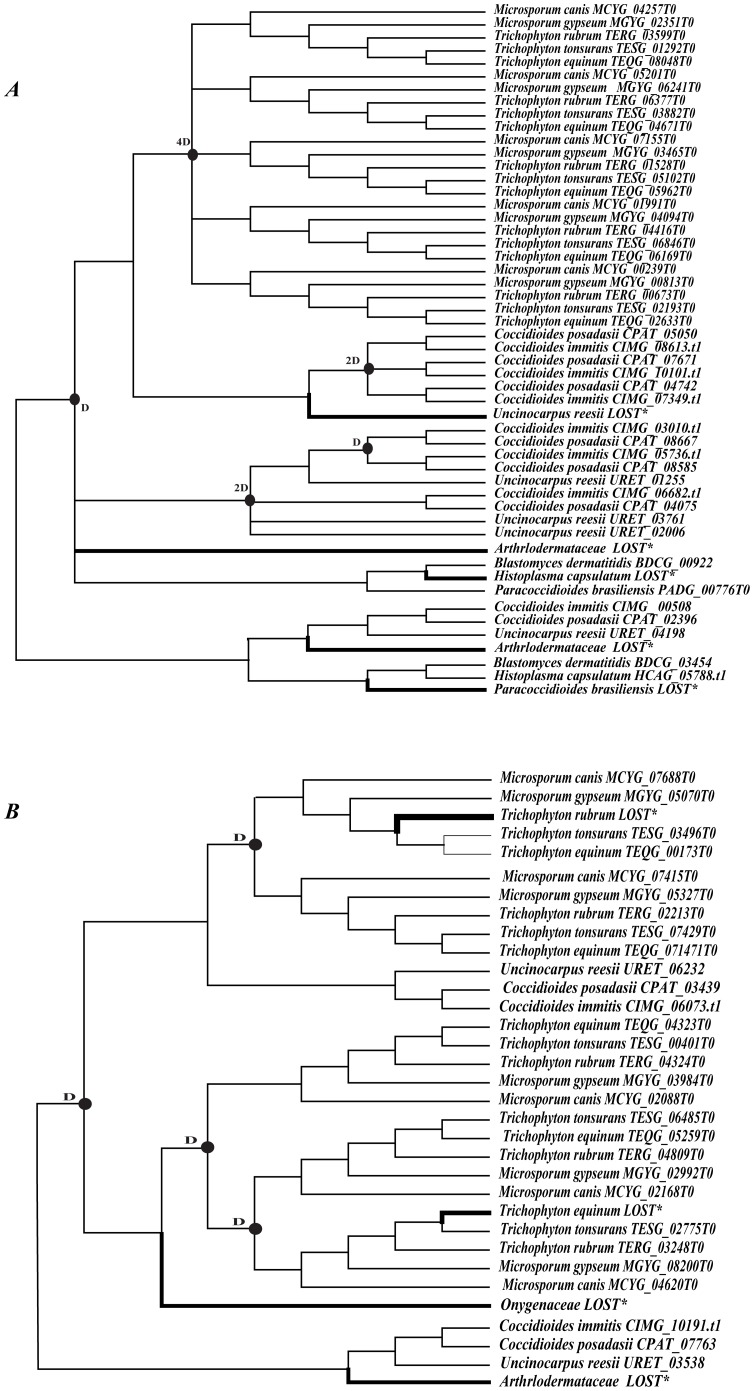
Duplication and loss events of M35 family genes and M36 family genes in Onygenales fungi. The reconciliation between species tree and gene tree of Onygenales fungi along with the confirmation of the gene loss/duplication scenario were determined by using Notung 2.6 [21]. The species tree of Onygenales fungi is shown as Fig. S7. Putative duplication events are indicated with solid cycles, while loss events are indicated with thick branches. a, duplication and loss events of M35 family genes. b, duplication and loss events of M36 family genes.

Based on the above results, we found that M35 and M36 family genes have undergone expansion in Onygenales fungi. To infer the gene duplication and loss scenarios of M35 and M36 family genes in Onygenales, We used Notung 2.6 [21], [22] to reconcile the species tree (Fig. S7) and the gene tree of each family. Using various tree-building methods, our inferred species tree consistently showed three strongly supported clades (Fig. S3), consistent with previous reports, which suggested that the fungal family Onygenaceae was more closely related to Arthodermataceae than to Ajellomycetace [13], [40]-[42]. The inferences of gene duplication and loss events in Onygenales species are shown in Figure 3.

For the M35 family genes (Figure 3a), there were at least 10 gene duplication and 5 gene loss events in the Onygenales species. An initial duplication event occurred before the divergence of Onygenales fungi. Subsequently, four and five gene duplications occurred independently in Arthodermataceae and Onygenaceae, respectively. In Onygenaceae, two gene duplications occurred before the divergence of *Coccidioides* and *U. reesii*, while three occurred before the divergence of *C. posadasii* and *C. immitis* (Figure 3a). For comparison, one M35 gene might have been lost in each of *U. reesii*, *P. brasiliensis* and *H. capsulatum*. Moreover, the lineages of Arthodermataceae might have also suffered two gene loss events after the duplication but before the divergence of the five dermatophytic fungi (Figure 3a).

For the M36 family genes (Figure 3b), there were four gene duplication and four gene loss events in Onygenales species. Before the divergence of Onygenales, there was one initial duplication event. Subsequently, three duplication events occurred in Arthodermataceae. There was one gene loss event in each of the two lineages of Onygenaceae and Arthodermataceae respectively and one gene was also lost in each of *T. equinum* and *T. rubrum*.

**Figure 4:**
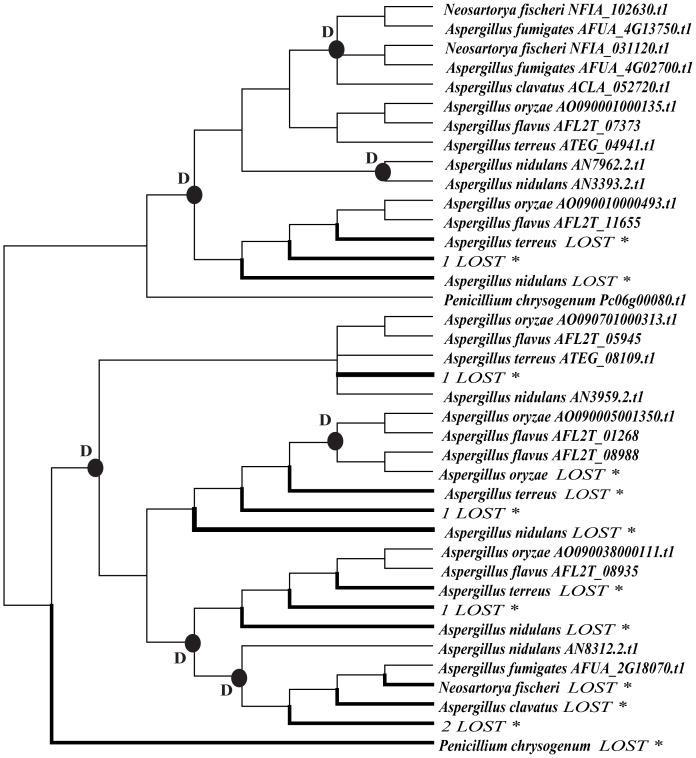
Duplication and loss events of M35 family genes in Eurotiales fungi. The reconciliation between species tree and gene tree of Eurotiales fungi along with the confirmation of the gene loss/duplication scenario were determined by using Notung 2.6 [21]. The species tree of Eurotiale fungi is shown as Fig. S8. Putative duplication events are indicated with solid cycles, while loss events are indicated with thick branches. 1, the lineage includes species of *A. fumigatus, N. fischeri* and *A. clavatus*. 2, the lineage includes species of *A. nidulans*, *A. terreus, A. oryza* and *A. flavus*.

In our study, we also found large variations of M35 family gene number in Eurotiales, ranging from 0 to 6 (Table 1). To infer the gene duplication and loss scenarios of M35 genes in this group, we also used Notung 2.6 [21], [22] to reconcile the species tree (Fig. S8) and the gene tree of this family. The inferences of gene duplication and loss events in Eurotiales species are shown in Figure 4. We found at least 7 gene duplication and 14 gene loss events in the Eurotiales species. While most of the duplication events occurred before the divergence of *Aspergillus* spp, the gene loss events in Eurotiales fungi were more dynamic. There were three gene loss events occurred in *Aspergillus nidulans* and *Aspergillus terreus* each, and one gene might have been lost in *Aspergillus oryzae, Aspergillus clavatus* and *Penicillium chrysogenum* each. Moreover, four gene loss events occurred in the lineage including three species (*A. fumigatus, Neosartorya fischeri* and *A. clavatus*) and one gene might have been lost before the divergence of the four species including *A. nidulans*, *A. terreus, A. oryza* and *Aspergillus flavus*.

### Selective pressure analyses in Onygenales

**Figure 5:**
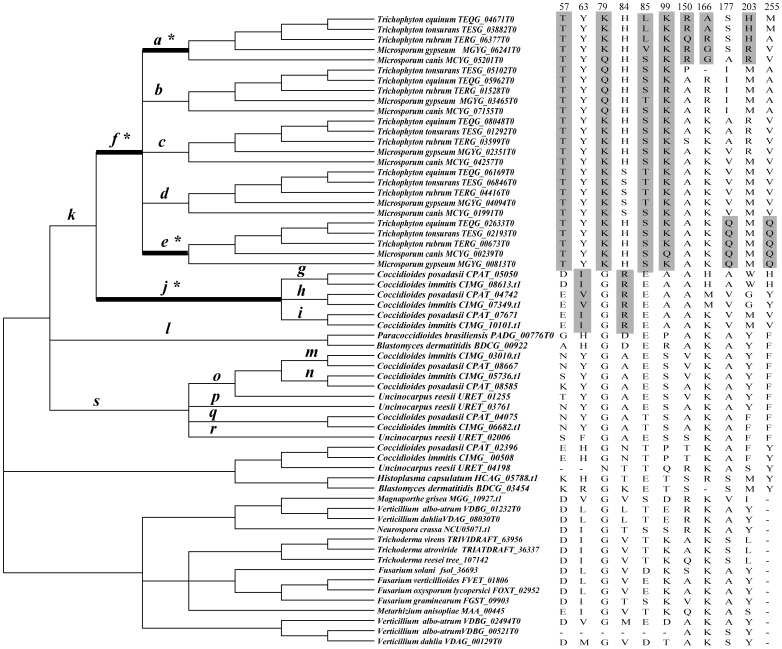
Phylogenetic tree of 62 M35 family genes used for codon-based maximum likelihood analysis in PAML. Phylogenetic trees were collapsed with inconsistent nodes from different tree-building methods and poor statistical supports into polytomy. Branches *a-s* indicated putative duplication events in Onygenales fungi. The branches with significant evidence of positive selection are indicated as a thick branch. The putative positively selected residues along these branches were shaded in grey.

Table 2 shows the evidence of positive selection analyses of M35 family genes in Onygenales. In the branch-specific model analyses [43], the free-ratio model, M1, revealed a significantly better fit to the data than did the one-ratio model, M0 (p<0.001, Table 3), suggesting that these M35 family genes have been the subjects of different selective pressures. In the site-specific model analyses, although the LRT of M2a/M1a did not achieve statistical significance (P = 1.000), M8, another positive-selection model provided a significantly better fit to the data than did the neutral model (M7) (P<0.001), suggesting the possibility of positive selection acting on the M35 family genes in Onygenales examined here. When we performed the branch-site model tests for those branches resulted from gene duplications of M35 family in Onygenales (19 branches in total, *a-t* as indicated in Figure 5), we found that there were five branches (branches *a*, *d, e, f* and *j*) showing signs of positive selection (Figure 5). After Bonferroni correction for multiple testing, we found that LRT tests were still significant in four of the branches (branch *a*, *e, f* and *j*; p<0.002632) (Table 2, Figure 5). Remarkably, several positively selected residues (A150R/Q, K166A/R/G and M203R/H for branch *a*, V177Q and Y255Q for branch *e*, E57T, G79K/Q, E85S/T/L and A99K for branch *f*, Y63V/I and A84R for branch *j*) were also identified for these branches with high posterior probabilities (Table 2 and Figure 5). Among these eleven positive selected sites, ten of them (A150R/Q, K166A/R/G, M203R/H, V177Q, E57T, G79K/Q, E85S/T/L, A99K, Y63V/I and A84R) were involved in amino acids substitutions with different physico-chemical properties (Table. S1).

**Table 2:** CODEML analyses of selective pattern for M35 family genes.

Models			In*L* a	Parameter Estimates	2Δ*L* b	Positively Selected Sites^c^
Branch-specific models		M0	−16572.48016	ω = 0.25237	455.838***	
		M1a	−16344.56115			
Site-specific models		M1a	−16214.65883	ω0 = 0.17007,ω1 = 1,p0 = 0.67270,p1 = 0.32730		Not allowed
		M2a	−16214.65883	ω0 = 0.17007,ω1 = 1,ω2 = 1,p0 = 0.67270,p1 = 0.23742,p2 = 0.08988		None
		M7	−15986.8697	p = 0.63889,q = 1.50238	425.426***	Not allowed
		M8	−16199.58278	p = 0.25117,q = 1.45893,p0 = 1.00000,p1 = 0.00000,ω = 2.85555		55(0.999), 76(0.983)
Branch-site models	Branch *a*	Null	−29907.40643	ω0 = 0.19365,ω1 = 1,ω2 = 1,p0 = 0.60899,p2a = 0.03351,p2b = 0.01865	12.5344***	150 (0.993), 166(0.991),203(0.995)
		Alterative	−29901.13922	ω0 = 0.19364,ω1 = 1,**ω2 = 36.77462**,p0 = 0.60444,p2a = 0.03748,p2b = 0.02091		
	Branch *e*	Null	−29906.27165	ω0 = 0.19306,ω1 = 1,ω2 = 1,p0 = 0.59293,p2a = 0.04993,p2b = 0.02774	30.5388***	177(0.998),255(0.997)
		Alterative	−29891.00224	ω0 = 0.19391,ω1 = 1,**ω2 = 44.75155**,p0 = 0.55743,p2a = 0.08606,p2b = 0.04768		
	Branch *f*	Null	−29901.87988	ω0 = 0.19307,ω1 = 1,ω2 = 1,p0 = 0.47997,p2a = 0.17002,p2b = 0.09155	37.969704***	57(0.995),79(0.997),85(0.997),99(0.991)
		Alterative	−29882.89503	ω0 = 0.19340,ω1 = 1,**ω2 = 98.15194**,p0 = 0.55311,p2a = 0.09909,p2b = 0.05284		
	Branch *j*	Null	−29907.74419	ω0 = 0.19363,ω1 = 1,ω2 = 1,p0 = 0.55036,p2a = 0.09431,p2b = 0.05198	9.352552***	63(0.983), 84(0.980)
		Alterative	−29903.06791	ω0 = 0.19389,ω1 = 1,**ω2 = 21.51283**,p0 = 0.61895,p2a = 0.02686,p2b = 0.01473		

a In*L* is the log-likelihood scores.

b LRT to detect adaptive evolution. *** P<0.001

c Posterior probabilities value of each codon site were showed in parentheses.

**Table 3:** CODEML analyses of selective pattern for M36 family genes.

Models			In*L* a	Parameter Estimates	2Δ*L* b	Positively Selected Sites^c^
Branch-specific models		M0	−20322.45718	ω = 0.14200	348.64117***	
		M1a	−20148.1366			
Site-specific models		M1a	−19897.27048	ω0 = 0.09291,ω1 = 1,p0 = 0.80509,p1 = 0.19491		Not allowed
		M2a	−19897.27048	ω0 = 0.09291,ω1 = 1,ω2 = 1,p0 = 0.80509,p1 = 0.13245,p2 = 0.06245		None
		M7	−19953.94322	p = 0.38777,q = 1.85670	472.167858***	Not allowed
		M8	−20190.02715	p = 1.04901,q = 1.52775,p0 = 1.00000,p1 = 0.00000,ω = 2.59980		158 (0.999), 172(0.999), 198(0.992), 208 (0.992), 251(0.998), 380 (0.996), 391(0.993)
Branch-site models	Branch *a*	Null	−19892.13564	ω0 = 0.09167,ω1 = 1,ω2 = 1,p0 = 0.72573,p2a = 0.08330,p2b = 0.01966	9.52853***	183 (0.996), 267(0.996),401(0.993)
		Alterative	−19887.37138	ω0 = 0.09239,ω1 = 1,**ω2 = 30.85099**,p0 = 0.79114,p2a = 0.01902,p2b = 0.00446		
	Branch *b*	Null	−19890.9925	ω0 = 0.09154,ω1 = 1,ω2 = 1,p0 = 0.73420,p2a = 0.07504,p2b = 0.01769	13.15426***	40(0.996)
		Alterative	−19884.41539	ω0 = 0.09212,ω1 = 1,**ω2 = 15.10889**,p0 = 0.78120,p2a = 0.03033,p2b = 0.00704		
	Branch *g*	Null	−19884.48335	ω0 = 0.09220,ω1 = 1,ω2 = 1,p0 = 0.68433,p2a = 0.13556,p2b = 0.02978	21.171318***	139(0.994), 221(0.995),232(0.996),300(0.990)510(0.998)
		Alterative	−19873.89769	ω0 = 0.09356,ω1 = 1,**ω2 = 11.57996**,p0 = 0.76788,p2a = 0.05883,p2b = 0.01233		

a In*L* is the log-likelihood scores.

b LRT to detect adaptive evolution. *** P<0.001

c Posterior probabilities value of each codon site were showed in parentheses.

**Figure 6:**
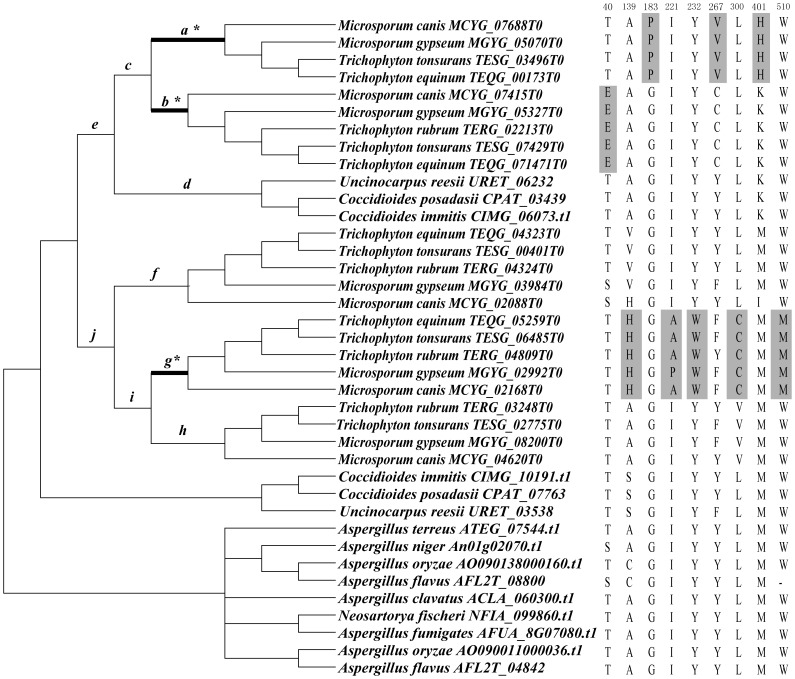
Phylogenetic tree of 38 M36 family genes used for codon-based maximum likelihood analysis in PAML. Phylogenetic trees were collapsed with inconsistent nodes from different tree-building methods and poor statistical supports into polytomy. Branches *a-j* indicated putative duplication events in Onygenales fungi. The branches with significant evidence of positive selection are indicated as a thick branch. The putative positively selected residues along these branches were shaded in grey.

Table 3 shows the results of positive selection analyses of M36 family genes in Onygenales. In the branch-specific model analyses [43], the free-ratio model, M1, revealed a significantly better fit to the data than did the one-ratio model, M0 (p<0.001, Table 3), suggesting that these M36 family genes have been the subjects of different selective pressures. Similar as the results in M35 family, only the LRT of M8/M7 achieved statistical significance (P<0.001) in the site-specific model analyses, also suggesting the possibility of positive selection acting on the M36 family genes in Onygenales examined here. Intriguingly, the LRT tests based on the branch-site models for those branches resulted from gene duplications of M36 family in Onygenales (12 branches in total, *a-l* as indicated in Figure 6) suggested that there was significant evidence along several lineages leading to the duplicated genes in dermatophytic fungi (branch *a*, *b* and *g*). After performing Bonferroni correction for multiple testing, the LRT tests still showed significant results in these branches (p<0.005). In addition, several residues (G183P, Y267V and K401H for branch *a*, T40E for branch *b*, A139H, I221A/P, Y232W, L300C and W510M for branch *g*) were predicted as positively selected sites along these branches (Table 3 and Figure 6). Among these nine positive selected sites, five of them (G183P, Y267V, T40E, A139H, Y232W) were involved in amino acid substitutions with different physico-chemical properties (Table. S1)

## Discussions

To get a comprehensive view of fungal M35 family and M36 family genes, we conducted genome-wide investigations and phylogenetic analyses of these two family genes from 50 sequenced Ascomycota fungal genomes with different life styles. Our results provided valuable insights for understanding the evolution of these two gene families in various fungi. In Sordariomycetes, the saprophytic fungi had 0 to 2 *Mep* genes, whereas the phytopathogenic fungi contained 2 to 6 genes within each species. Phylogenetic analyses suggested that the M35 family genes of Sordariomycetes fungi fell into two clades: *M35-i* and *M35-l*. Clade *M35-l* contained the genes from Sordariomycetes that included saprophytic, phytopathogenic and entomopathogenic fungi, while clade *M35-I* comprised six duplicated genes from six phytopathogenic fungi each (Figure 1). In addition, the M36 family genes in Sordariomycetes fungi also grouped into two clades: *M36-7* and *M36-8*, with *M36-8* containing the duplicated genes from the two phytopathogenic fungi *V. albo-atrum* and *V. dahlia* (Figure 2). Similarly, M35 family genes from the three Leotomycetes fungi (*Botryotinia fuckeliana*, *Botrytis cinerea* and *Sclerotinia sclerotiorum*) were also clustered into two clades: *M35-j* and *M35-k*, indicating that the genes have duplicated before the divergence of these three phytopathogenic fungi. Previous studies suggested that protease gene family expansion appeared to be an important evolutionary step among the phytopathogenic fungi and likely served important roles in plant infection [44]. We speculate that the duplication of *Mep* genes occurred in the phytopathogenic fungi belonging to Sordariomycetes and Leotomycetes may be associated with their pathogenicity. However, we did not identify any *Mep* gene from the two endophytic fungi, *Chaetomium globosum* and *Epichloë festucae*, and no M36 family gene was identified from the three Leotomycetes fungi, suggesting that these genes may be lost during the evolution of these fungi.

Based on the homologous search results, large variations of gene number, ranging from 1 to 8 were also observed in Eurotiales (Table 1). Phylogenetic analyses and gene duplication/loss analyses suggested that the *Mep* genes, especially the M35 family genes have been highly dynamic through the evolution of Eurotiales fungi (Figure 1, 2 and 4). In our study, *A. oryzae*, which is a saprophytic fungus and plays a key role in the fermentation process of several traditional Japanese beverages and sauces [45], contains 7 *Meps* in total (5 in M35 family and 2 in M36 family), whereas *A. fumigatus*, an important opportunistic pathogen of vertebrates with compromised immune systems [46], has only 4 Mep genes in total (3 in M35 family and 1 in M36 family). However, although *A. fumigatus* contains fewer Mep genes, it has been reported that this fungus can utilize the M36 family metalloprotease to help it invade mammalian lung and degrade its host's proteinaceous structural barriers [9]. Moreover, other metalloproteases, such as Aspf2 from *A. fumigatus* and Aspnd1 from *A. nidulans* which are known cell-surface antigens during fungal infections and can bind specific ligands within mammalian hosts, were also previously identified from *Aspergillus* spp. [47]. Therefore, we believed that the opportunistic human pathogens in Eurotiales might not only use the M35 or M36 family genes as virulence factors, but also choose other families of metalloproteases as pathogenic factors to carry out the infection of immunocompromised individuals.

Surprisingly, neither M35 gene nor M36 gene was found in Saccharomycotina and Taphrinomycotina fungi, suggesting that both the two family genes have been lost during the evolution of the yeast-like fungi. However, although no M35 or M36 family proteins have been identified in yeast-like fungi, other types of metalloproteases that serve important roles in the development and pathogenicity of yeast-like fungi have been identified before [48]. For example, a Zps1p metalloproteases which involved in zinc-responsive transcriptional regulation was observed in *Saccharomyces cerevisiae* [49]. Moreover, a Zps1p-like metalloproteases named Pra1 which function as virulence factors in mediating critical host-parasite interactions were identified from the opportunistic human pathogen *Candida albicans* that can cause diseases of vertebrates [48]. Sequence alignments suggested that some critical residues such as the cysteine residues involved in disulfide bond formation and a zinc-binding domain were conserved in both Zps1p and M35 family metalloproteases [48]. These results suggest that the yeast-like fungi may utilize other metalloproteases but not M35 or M36 family proteins to carry out their development and pathogenicity.

The most striking result from our study is the significant varieties of *Meps* in Onygenales fungi. Phylogenetic reconstruction and gene duplication/loss analyses suggest that the first duplication event of both M35 and M36 family genes occurred in the common ancestor of the Onygenales species (Figure 1, 2 and 3). Subsequently, additional gene duplication events mainly occurred after the divergence of Arthrlodermataceae (dermatophyte) and Onygenaceae species (Figure 1, 2 and 3), leading to a large number of Mep genes shared between Arthrlodermataceae (dermatophyte) and Onygenaceae species. However, the Ajellomycetace species (*B. dermatitidis*, *H. capsulatum* and *P. brasiliensis*) did not show evidence of expansion of these genes (only containing 1 or 2 M35 family genes and no M36 family gene was identified from them). Although the species in Onygenales showed a host/substrate shift from plants to animals during evolution, their pathogenicity factors on animals might be different. Dermatophytic fungi cause the majority of superficial dermatophytosis in humans and animals [12], while *Coccidioides* spp. can cause life-threatening respiratory disease known as coccidioidomycosis (Valley fever) in immunocompromised individuals [50], [51]. However, Ajellomycetace species cause geographically widespread systemic mycosis of humans and other mammals [52]. For example, *Paracoccidioides* can cause paracoccidioidomycosis [40], *B. dermatitidis* is the causative agent of blastomycosis. However, *H. capsulatum* is the most common cause of histoplasmosis in the world [53]. Therefore, the distinct expansion of Meps in Arthrlodermataceae and Onygenaceae species suggest that the Meps may have played different roles during the evolution of dermatophytic and Onygenaceae species, providing flexibility for these species to occupy different ecological niches.

Interestingly, LRT analysis in both M35 family and M36 family suggested that the duplicated *Meps* genes most likely underwent positive selection during the evolution in dermatophytic and *Coccidioides* species. For dermatophytic fungi, significantly different selective pressures have acted on several lineages leading to both duplicated M35 family (branches *a*, *e* and *f*) (Figure 5) and M36 family genes (branches *a*, *b* and *g*) (Figure 6). The results suggested that functional divergences of both M35 family and M36 family genes in dermatophytic fungi most likely occurred after gene duplication and before the speciation of these five dermatophytic species. The functional adaptations of these duplicated Meps in dermatophytic fungi might have helped these fungi survive in various niches because dermatophytes have many different environmental niches (soil, skin, hair), with different hosts (plants and animals), and with different cohabitating microbes. The characterization and identification of several Mep's functions in recent studies further indicated the important functions of Meps during host adaptation in dermatophytic fungi. For example, a 43.5-kDa Meps belonging to the M35 family in the dermatophytic fungus *M. canis* was identified and it could digest keratin azure during infection of cats and guinea pigs [3]. However, the expression of genes encoding M36 fungalysins in *A. benhamiae* assayed by cDNA microarray analysis suggested that the M36 family genes not only help *A. benhamiae* digesting keratin, but also facilitate the cutaneous infection of guinea pigs [54]. Both the M35 family and M36 family genes have the capacity to degrade keratin, suggesting that the cooperation between the two family genes may have facilitated the fungi to adapt to various environmental niches.

For Onygenaceae fungi, the positive selection pressure might have acted on the ancestral lineage (branch *j*) (Figure 5) leading to the three additional duplicated genes specifically in *Coccidioides* species, consistent with the results of our earlier study which suggested that significant selective pressure on the ancestral lineage of three additional duplicated M35 family genes from *Coccidioides* species may be associated with recent pathogenesis acquisition of M35 family genes in the *Coccidioides* species [13]. Noticeably, although the M36 family genes from *Coccidioides* species and *U. reesii* were duplicated before the split of Arthrlodermataceae and Onygenaceae species, positive selection did not seem to have acted on the duplicated M36 family genes in Onygenaceae species. These results suggest that the M36 family genes in *Coccidioides* species and *U. reesii* are unlikely important functional genes during the evolution of these fungi. Of course, we can't exclude the possibility that these M36 family genes in *Coccidioides* and *U. reesii* may serve other yet unknown physiological functions.

In our study, eleven and nine amino acid substitutions were predicted as positively selected sites respectively for the M35 and M36 family genes in Onygenales fungi (Table 2 and 3, Figure 5 and 6). Most of the positively selected sites had substitutions of amino acids with different physical-chemically properties, such as differences in polarity, charge and/or hydrophobicity (Table. S1). It has been recognized that substitutions with chemically very different amino acids often have significant effects on the overall flexibility, structure stability and/or activity of proteins [55], [56]. Therefore, although the explicit functional changes of the Meps brought by these positive selected residues identified in our study cannot be predicted at present, we propose that these residues may have allowed the development of new physiological functions of the duplicated *Mep* genes, providing valuable information for functional adaption of the duplicated Meps in dermatophytic fungi and *Coccidioides* species. Experimental investigations are needed in order test the functional effects of the potentially adaptive amino acid replacements discovered by our analysis.

## Conclusions

In summary, our study analyzed the phylogenetic relationships among genes in both M35 and M36 families from Ascomycota fungi. Our results increase the current knowledge of the evolution of Meps in fungi and support the possibility that crucial physiological functions of both duplicated M35 family and M36 family genes may have been developed during the evolution of fungi. Furthermore, our detection of significant positive selections in several lineages of dermatophytes and *Coccidioides* species indicates that rapid functional divergence of duplicated Meps have most likely occurred after gene duplications in these fungi. These species-specific adaptations could be important in host immune system interaction or in their survival in the environment. The hypotheses proposed in our analysis will provide valuable information for functional analysis of these genes in future studies.

## Supporting Information

Figure S1 **Amino acid sequences alignment for 62 M35 family genes with MUSCLE 3.5.**
(DOC)

Figure S2 **Amino acid sequences alignment for 38 M36 family genes with MUSCLE 3.5.**
(DOC)

Figure S3 **Amino acid sequences alignment of 105 M35 genes with MUSCLE 3.5.**
(DOC)

Figure S4 **The Bayesian tree based on 105 M35 family genes by using MrBayes 3.1.2.**
(DOCX)

Figure S5 **Amino acid sequences alignment of 58 M36 genes with MUSCLE 3.5.**
(DOC)

Figure S6 **The Bayesian tree based on 58 M36 family genes by using MrBayes 3.1.2.**
(DOCX)

Figure S7 **Species tree of Onygenales fungi used for gene duplication and loss analyses in the study.**
(DOCX)

Figure S8 **Species tree of Eurotiales fungi used for gene duplication and loss analyses in the study.**
(DOCX)

Table S1 **The physic-chemical properties of the positive selected amino acids identified in this study.**
(DOCX)

## References

[1] Soanes D, Alam I, Cornell M, Wong H, Hedeler C, et al (2008) Comparative genome analysis of filamentous fungi reveals gene family expansions associated with fungal pathogenesis. PLoS One 3: e2300. 18523684 10.1371/journal.pone.0002300 PMC2409186

[2] Monod M, Capoccia S, Léchenne B, Zaugg C, Holdom M, et al (2002) Secreted proteases from pathogenic fungi. International Journal of Medical Microbiology 292: 405–419. 12452286 10.1078/1438-4221-00223

[3] Brouta F, Descamps F, Monod M, Vermout S, Losson B, et al (2002) Secreted metalloprotease gene family of *Microsporum canis* . Infection and Immunity 70: 5676–5683. 12228297 10.1128/IAI.70.10.5676-5683.2002 PMC128366

[4] St Leger RJ, Bidochka MJ, Roberts DW (1994) Isoforms of the Cuticle-Degrading Pr1 Proteinase and Production of a Metalloproteinase by *Metarhizium anisopliae* . Archives of Biochemistry and Biophysics 313: 1–7. 8053668 10.1006/abbi.1994.1350

[5] Gillespie JP, Bailey AM, Cobb B, Vilcinskas A (2000) Fungi as elicitors of insect immune responses. Archives of Insect Niochemistry and Physiology 44: 49–68. 10.1002/1520-6327(200006)44:2<49::AID-ARCH1>3.0.CO;2-F 10861866

[6] Jia Y, McAdams SA, Bryan GT, Hershey HP, Valent B (2000) Direct interaction of resistance gene and avirulence gene products confers rice blast resistance. The EMBO Journal 19: 4004–4014. 10921881 10.1093/emboj/19.15.4004 PMC306585

[7] Rawlings ND, Morton FR, Barrett AJ (2006) MEROPS: the peptidase database. Nucleic Acids Research 34: D270. 16381862 10.1093/nar/gkj089 PMC1347452

[8] Hori T, Kumasaka T, Yamamoto M, Nonaka T, Tanaka N, et al (2001) Structure of a new ‘aspzincin’ metalloendopeptidase from Grifola frondosa: implications for the catalytic mechanism and substrate specificity based on several different crystal forms. Acta Crystallographica Section D: Biological Crystallography 57: 361–368. 11223512 10.1107/s0907444900019740

[9] Markaryan A, Morozova I, Yu H, Kolattukudy PE (1994) Purification and characterization of an elastinolytic metalloprotease from *Aspergillus fumigatus* and immunoelectron microscopic evidence of secretion of this enzyme by the fungus invading the murine lung. Infection and Immunity 62: 2149–2157. 8188335 10.1128/iai.62.6.2149-2157.1994 PMC186491

[10] Mathy A, Baldo A, Schoofs L, Cambier L, Defaweux V, et al (2010) Fungalysin and dipeptidyl-peptidase gene transcription in *Microsporum canis* strains isolated from symptomatic and asymptomatic cats. Veterinary Microbiology 146: 179–182. 20488631 10.1016/j.vetmic.2010.04.019

[11] Sharpton T, Stajich J, Rounsley S, Gardner M, Wortman J, et al (2009) Comparative genomic analyses of the human fungal pathogens *Coccidioides* and their relatives. Genome Research 19: 1722–1731. 19717792 10.1101/gr.087551.108 PMC2765278

[12] Burmester A, Shelest E, Glöckner G, Heddergott C, Schindler S, et al (2011) Comparative and functional genomics provide insights into the pathogenicity of dermatophytic fungi. Genome Biology 12: R7. 21247460 10.1186/gb-2011-12-1-r7 PMC3091305

[13] Li J, Yu L, Tian Y, Zhang KQ (2012) Molecular Evolution of the Deuterolysin (M35) Family Genes in *Coccidioides* . PLoS One 7: e31536. 22363666 10.1371/journal.pone.0031536 PMC3282736

[14] Edgar R (2004) MUSCLE: multiple sequence alignment with high accuracy and high throughput. Nucleic Acids Research 32: 1792–1797. 15034147 10.1093/nar/gkh340 PMC390337

[15] Castresana J (2000) Selection of conserved blocks from multiple alignments for their use in phylogenetic analysis. Molecular Biology and Evolution 17: 540–552. 10742046 10.1093/oxfordjournals.molbev.a026334

[16] Talavera G, Castresana J (2007) Improvement of phylogenies after removing divergent and ambiguously aligned blocks from protein sequence alignments. Systematic Biology 56: 564–577. 17654362 10.1080/10635150701472164

[17] Guindon S, Gascuel O (2003) A simple, fast, and accurate algorithm to estimate large phylogenies by maximum likelihood. Systematic Biology 52: 696–704. 14530136 10.1080/10635150390235520

[18] Ronquist F, Huelsenbeck J (2003) MrBayes 3: Bayesian phylogenetic inference under mixed models. Bioinformatics 19: 1572–1574. 12912839 10.1093/bioinformatics/btg180

[19] Abascal F, Zardoya R, Posada D (2005) ProtTest: selection of best-fit models of protein evolution. Bioinformatics 21: 2104–2105. 15647292 10.1093/bioinformatics/bti263

[20] Felsenstein J (1985) Confidence limits on phylogenies: an approach using the bootstrap. Evolution 39: 783–791. 28561359 10.1111/j.1558-5646.1985.tb00420.x

[21] Chen K, Durand D, Farach-Colton M (2000) NOTUNG: a program for dating gene duplications and optimizing gene family trees. Journal of Computational Biology 7: 429–447. 11108472 10.1089/106652700750050871

[22] Durand D, Halldórsson BV, Vernot B (2006) A hybrid micro-macroevolutionary approach to gene tree reconstruction. Journal of Computational Biology 13: 320–335. 16597243 10.1089/cmb.2006.13.320

[23] James TY, Kauff F, Schoch CL, Matheny PB, Hofstetter V, et al (2006) Reconstructing the early evolution of Fungi using a six-gene phylogeny. Nature 443: 818–822. 17051209 10.1038/nature05110

[24] Akaike H (1974) A new look at the statistical model identification. Automatic Control, IEEE Transactions on 19: 716–723.

[25] Posada D, Buckley TR (2004) Model selection and model averaging in phylogenetics: advantages of Akaike information criterion and Bayesian approaches over likelihood ratio tests. Systematic Biology 53: 793–808. 15545256 10.1080/10635150490522304

[26] Posada D, Crandall KA (1998) MODELTEST: testing the model of DNA substitution. Bioinformatics 14: 817–818. 9918953 10.1093/bioinformatics/14.9.817

[27] Lewis PO, Holder MT, Holsinger KE (2005) Polytomies and Bayesian phylogenetic inference. Systematic Biology 54: 241–253. 16012095 10.1080/10635150590924208

[28] Hurst LD (2002) The Ka/Ks ratio: diagnosing the form of sequence evolution. Trends in Genetics 18: 486–487. 12175810 10.1016/s0168-9525(02)02722-1

[29] Bielawski JP, Yang Z (2004) A maximum likelihood method for detecting functional divergence at individual codon sites, with application to gene family evolution. Journal of Molecular Evolution 59: 121–132. 15383915 10.1007/s00239-004-2597-8

[30] Bielawski J, Yang Z (2003) Maximum likelihood methods for detecting adaptive evolution after gene duplication. Journal of Structural and Functional Genomics 3: 201–212. 12836699

[31] Yang Z (2007) PAML 4: phylogenetic analysis by maximum likelihood. Molecular Biology and Evolution 24: 1586–1591. 17483113 10.1093/molbev/msm088

[32] Suzuki Y, Nei M (2001) Reliabilities of parsimony-based and likelihood-based methods for detecting positive selection at single amino acid sites. Molecular Biology and Evolution 18: 2179–2185. 11719567 10.1093/oxfordjournals.molbev.a003764

[33] Yang Z (1998) Likelihood ratio tests for detecting positive selection and application to primate lysozyme evolution. Molecular Biology and Evolution 15: 568–573. 9580986 10.1093/oxfordjournals.molbev.a025957

[34] Yang Z, Wong WSW, Nielsen R (2005) Bayes empirical Bayes inference of amino acid sites under positive selection. Molecular Biology and Evolution 22: 1107–1118. 15689528 10.1093/molbev/msi097

[35] Gillespie J (1991) The Causes of Molecular Evolution: Oxford University Press, New York.

[36] Zhang J, Nielsen R, Yang Z (2005) Evaluation of an improved branch-site likelihood method for detecting positive selection at the molecular level. Molecular Biology and Evolution 22: 2472–2479. 16107592 10.1093/molbev/msi237

[37] Nielsen R, Yang Z (1998) Likelihood models for detecting positively selected amino acid sites and applications to the HIV-1 envelope gene. Genetics 148: 929–936. 9539414 10.1093/genetics/148.3.929 PMC1460041

[38] Bonferroni C (1935) Il calcolo delle assicurazioni su gruppi di teste. Studi in Onore del Professore Salvatore Ortu Carboni 13.

[39] Bonferroni CE (1936) Teoria statistica delle classi e calcolo delle probabilita: Libreria internazionale Seeber.

[40] Desjardins CA, Champion MD, Holder JW, Muszewska A, Goldberg J, et al (2011) Comparative genomic analysis of human fungal pathogens causing paracoccidioidomycosis. PLoS Genetics 7: e1002345. 22046142 10.1371/journal.pgen.1002345 PMC3203195

[41] Ebersberger I, de Matos Simoes R, Kupczok A, Gube M, Kothe E, et al (2012) A consistent phylogenetic backbone for the fungi. Molecular Biology and Evolution 29: 1319–1334. 22114356 10.1093/molbev/msr285 PMC3339314

[42] Marcet-Houben M, Gabaldón T (2009) The tree versus the forest: the fungal tree of life and the topological diversity within the yeast phylome. PLoS One 4: e4357. 19190756 10.1371/journal.pone.0004357 PMC2629814

[43] Yang Z (1997) PAML: a program package for phylogenetic analysis by maximum likelihood. Computer Applications in the Biosciences 13: 555–556. 9367129 10.1093/bioinformatics/13.5.555

[44] Soanes DM, Alam I, Cornell M, Wong HM, Hedeler C, et al (2008) Comparative genome analysis of filamentous fungi reveals gene family expansions associated with fungal pathogenesis. PLoS One 3: e2300. 18523684 10.1371/journal.pone.0002300 PMC2409186

[45] Machida M, Asai K, Sano M, Tanaka T, Kumagai T, et al (2005) Genome sequencing and analysis of *Aspergillus oryzae* . Nature 438: 1157–1161. 16372010 10.1038/nature04300

[46] Nierman WC, Pain A, Anderson MJ, Wortman JR, Kim HS, et al (2005) Genomic sequence of the pathogenic and allergenic filamentous fungus *Aspergillus fumigatus* . Nature 438: 1151–1156. 16372009 10.1038/nature04332

[47] Segurado M, López-Aragón R, Calera JA, Fernández-Abalos JM, Leal F (1999) Zinc-regulated biosynthesis of immunodominant antigens from *Aspergillus* spp. Infection and Immunity 67: 2377–2382. 10225898 10.1128/iai.67.5.2377-2382.1999 PMC115981

[48] Sentandreu M, Elorza MV, Sentandreu R, Fonzi WA (1998) Cloning and characterization of PRA1, a gene encoding a novel pH-regulated antigen of Candida albicans. Journal of Bacteriology 180: 282–289. 9440517 10.1128/jb.180.2.282-289.1998 PMC106883

[49] Zhao H, Eide DJ (1997) Zap1p, a metalloregulatory protein involved in zinc-responsive transcriptional regulation in *Saccharomyces cerevisiae.* . Molecular and Cellular Biology 17: 5044–5052. 9271382 10.1128/mcb.17.9.5044 PMC232355

[50] Galgiani JN (1999) Coccidioidomycosis: a regional disease of national importance: rethinking approaches for control. Annals of Internal Medicine 130: 293–300. 10068388 10.7326/0003-4819-130-4-199902160-00015

[51] Hector R, Laniado-Laborin R (2005) Coccidioidomycosis—a fungal disease of the Americas. PLoS Medicine 2: e2. 15696207 10.1371/journal.pmed.0020002 PMC545195

[52] Untereiner W, Scott J, Naveau F, Sigler L, Bachewich J, et al (2004) The *Ajellomycetaceae*, a new family of vertebrate-associated Onygenales. Mycologia 96: 812. 21148901 10.1080/15572536.2005.11832928

[53] Durkin M, Kohler S, Schnizlein-Bick C, LeMonte A, Connolly P, et al (2001) Chronic infection and reactivation in a pulmonary challenge model of histoplasmosis. Journal of Infectious Diseases 183: 1822–1824. 11372039 10.1086/320720

[54] Staib P, Zaugg C, Mignon B, Weber J, Grumbt M, et al (2010) Differential gene expression in the pathogenic dermatophyte *Arthroderma benhamiae* in vitro versus during infection. Microbiology 156: 884–895. 19942661 10.1099/mic.0.033464-0

[55] Haydon DT, Bastos AD, Knowles NJ, Samuel AR (2001) Evidence for positive selection in foot-and-mouth disease virus capsid genes from field isolates. Genetics 157: 7–15. 11139487 10.1093/genetics/157.1.7 PMC1461471

[56] Sainudiin R, Wong WSW, Yogeeswaran K, Nasrallah JB, Yang Z, et al (2005) Detecting site-specific physicochemical selective pressures: applications to the Class I HLA of the human major histocompatibility complex and the SRK of the plant sporophytic self-incompatibility system. Journal of Molecular Evolution 60: 315–326. 15871042 10.1007/s00239-004-0153-1

