# Supplementary material for: Independent Expansion of Zincin Metalloproteinases in Onygenales Fungi May Be Associated with Their Pathogenicity
Source: PLoS One. 2014 Feb 28;9(2):e90225. doi: 10.1371/journal.pone.0090225 (PMC3938660; doi:10.1371/journal.pone.0090225)

**Figure S4. The Bayesian tree based on 105 M35 family genes** **by using MrBayes 3.1.2**

The tree was constructed using MrBayes 3.1.2 [[18](#_ENREF_18)]. The best-fitting model WAG+I+G and their parameters (I=0.03, G=1.912) which chosen by ProtTest [[19](#_ENREF_19)] were used in the priors of Bayesian inference. Bayesian analysis started with randomly generated trees and Metropolis-coupled Markov chain Monte Carlo (MCMC) analyses were run for 2×10^6^ generations. The run was stoped when the average standard deviation of split frequencies was less than 0.01 in all the cases (MrBayes 3.1.2 manual). To ensure that these analyses were not trapped in local optima, the dataset was run three times independently. We determined the burn-in period by checking for likelihood stability. A 50% majority rule consensus of post burn-in trees was constructed to summarize posterior probabilities (PPs) for each branch.


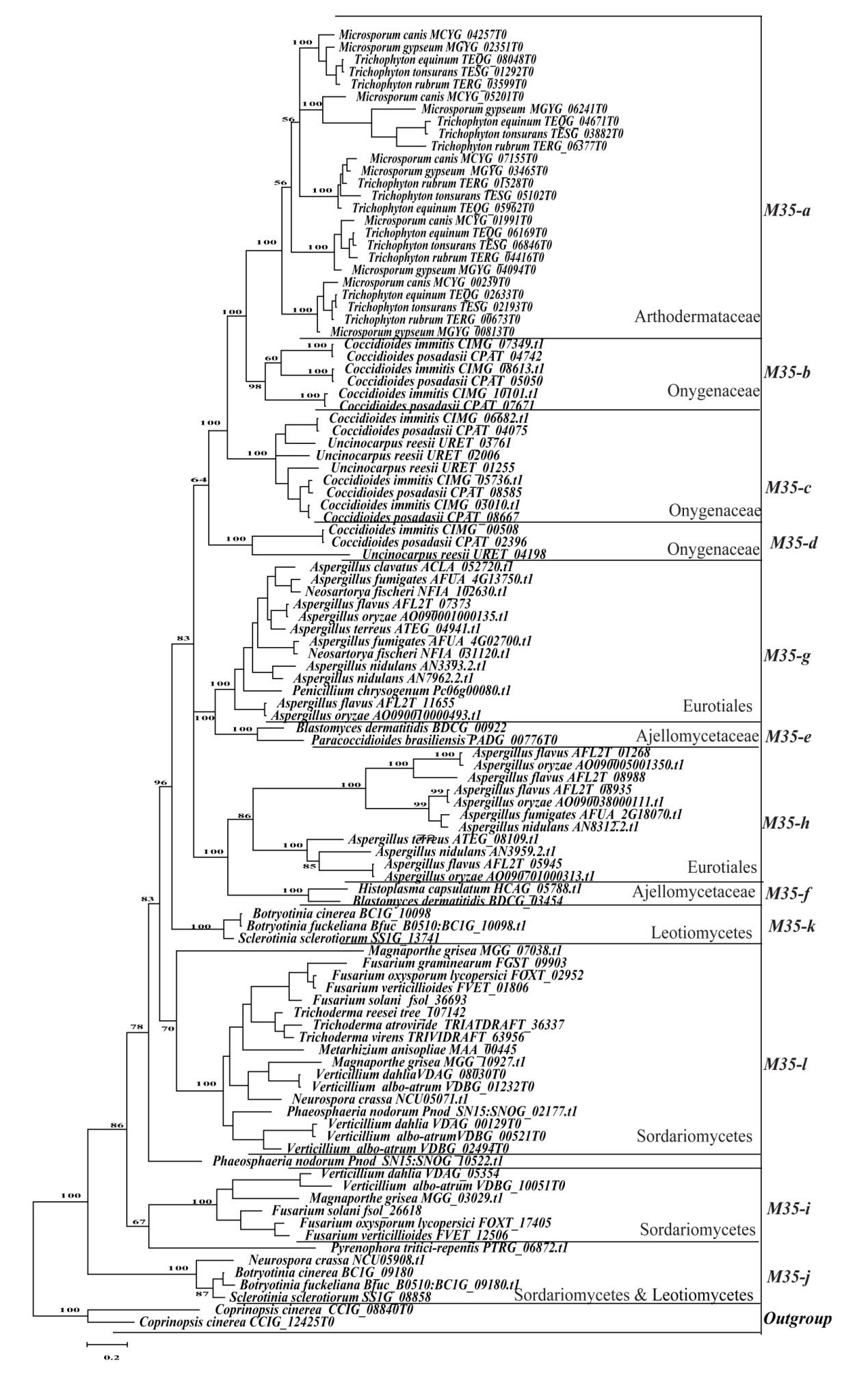

Supplement: Figure S4 — The Bayesian tree based on 105 M35 family genes by using MrBayes 3.1.2. (DOCX) [file pone.0090225.s004.docx]
