# Supplementary material for: Independent Expansion of Zincin Metalloproteinases in Onygenales Fungi May Be Associated with Their Pathogenicity
Source: PLoS One. 2014 Feb 28;9(2):e90225. doi: 10.1371/journal.pone.0090225 (PMC3938660; doi:10.1371/journal.pone.0090225)

**Figure S7. Species tree of Onygenales fungi used for gene duplication and loss analyses in the study.**

The species tree of Onygenales fungi was inferred by performing different tree-building methods from a combined alignment of six genes as those used in James et al [23], including 18S rRNA, 28S rRNA, ITS RNA, translation elongation factor 1-α (TEF1α), RNA polymerase II largest subunit (RPB1) and RNA polymerase II second largest subunit (RPB2).


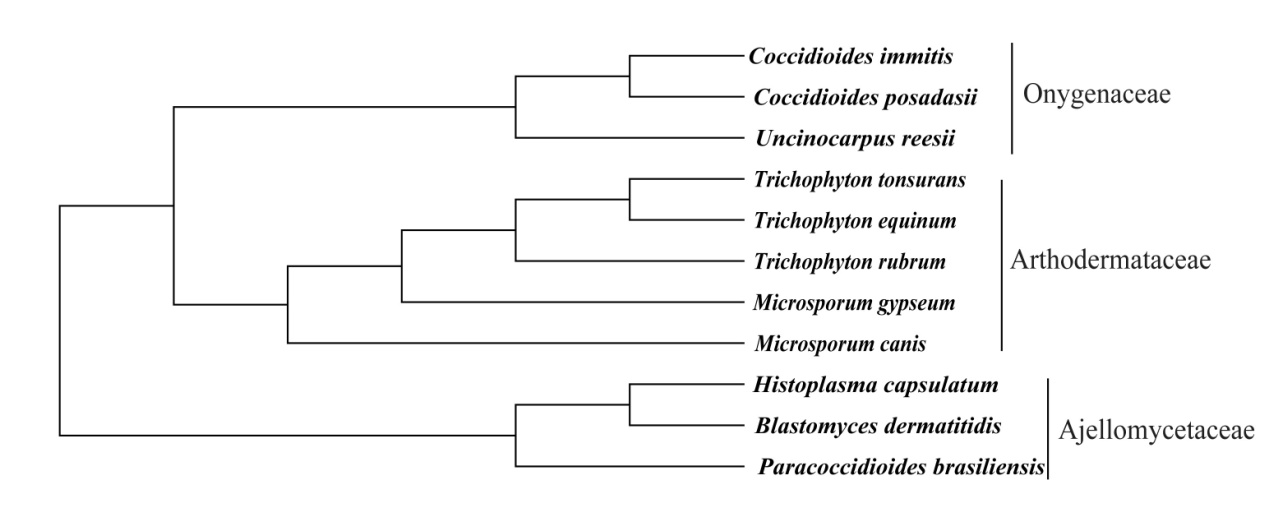

Supplement: Figure S7 — Species tree of Onygenales fungi used for gene duplication and loss analyses in the study. (DOCX) [file pone.0090225.s007.docx]
