# Supplementary material for: Independent Expansion of Zincin Metalloproteinases in Onygenales Fungi May Be Associated with Their Pathogenicity
Source: PLoS One. 2014 Feb 28;9(2):e90225. doi: 10.1371/journal.pone.0090225 (PMC3938660; doi:10.1371/journal.pone.0090225)

**Figure S8. Species tree of Eurotiales fungi used for gene duplication and loss analyses in the study.**

The species tree of Eurotiales fungi was inferred by using the same methods described in Figure S3.


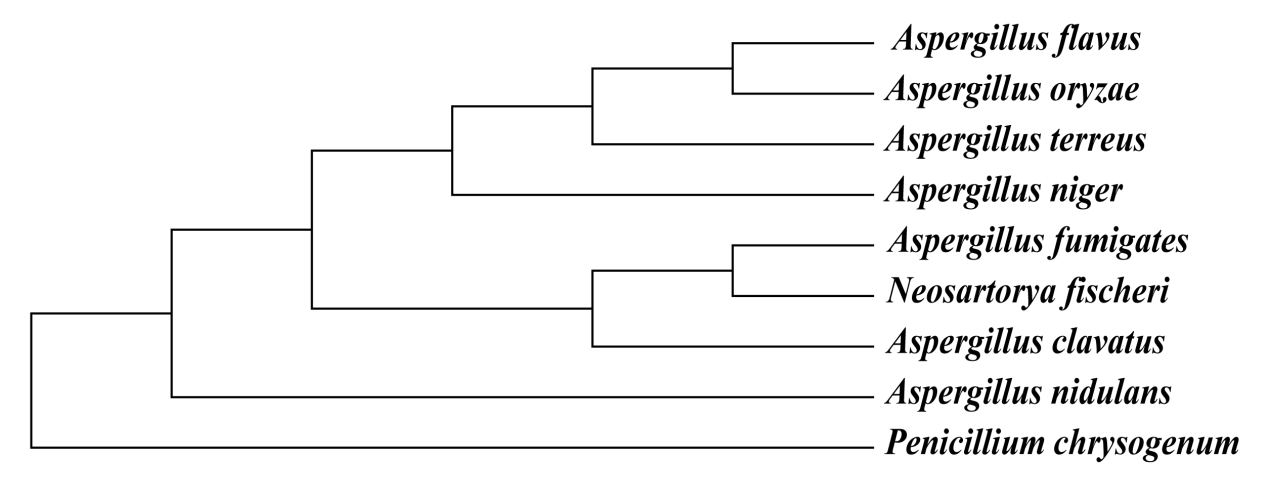

Supplement: Figure S8 — Species tree of Eurotiales fungi used for gene duplication and loss analyses in the study. (DOCX) [file pone.0090225.s008.docx]
