# Supplementary material for: Independent Expansion of Zincin Metalloproteinases in Onygenales Fungi May Be Associated with Their Pathogenicity
Source: PLoS One. 2014 Feb 28;9(2):e90225. doi: 10.1371/journal.pone.0090225 (PMC3938660; doi:10.1371/journal.pone.0090225)
Supplement: Table S1 — The physic-chemical properties of the positive selected amino acids identified in this study. (DOCX) [file pone.0090225.s009.docx]

**Table S1:** The physicochemical properties of the positive selected amino acids identified in this study.

|  | branches | site | original amino acid | physicochemical properties of original amino acid | substituted amino acid | physicochemical properties of substituted amino acid |
| --- | --- | --- | --- | --- | --- | --- |
| M35 family | *branch a* | 150 | A | nonpolar, neutral, hydrophobic | R/Q | R: basic polar, positive, alkaline; Q: polar, neutral |
|  |  | 166 | K | basic polar, positive, alkaline | A/R/G | A: hydrophobic R:basic polar, positive, alkaline; G:nonpolar, neutral |
|  |  | 203 | M | nonpolar, neutral, hydrophobic | R/H | R:basic polar, positive, alkaline; H:basic polar, positive/neutral,alkaline |
|  | *branch e* | 177 | V | nonpolar, neutral, hydrophobic | Q | polar, neutral |
|  |  | 255 | Y | polar, neutral | Q | polar, neutral |
|  | *branch f* | 57 | E | acidic, negative | T | polar, neutral |
|  |  | 79 | G | nonpolar, neutral | K/Q | K:basic polar, positive, alkaline; Q:polar, neutral |
|  |  | 85 | E | acidic, negative | S/T/L | S:polar,neutral; T: polar, neutral ; L: nonpolar, neutral |
|  |  | 99 | A | nonpolar, neutral, hydrophobic | K/Q | K: basic polar, positive, alkaline, positive ; Q: polar, neutral |
|  | *branch j* | 63 | Y | polar, neutral | V/I | V: nonpolar, neutral, hydrophobic |
|  |  | 84 | A | nonpolar, neutral, hydrophobic | R | basic polar, positive, alkaline |
| M36 family | *branch a* | 183 | G | nonpolar, neutral | P | nonpolar, neutral, hydrophobic |
|  |  | 267 | Y | polar, neutral | V/I | V: nonpolar, neutral, hydrophobic; I:nonpolar, neutral |
|  |  | 401 | K | basic polar, positive, alkaline | H | basic polar, positive/neutral,alkaline |
|  | *branch b* | 40 | T | polar, neutral | E | acidic, negative |
|  | *branch g* | 139 | A | nonpolar, neutral, hydrophobic | H | basic polar, positive/neutral,alkaline |
|  |  | 221 | I | nonpolar, neutral, hydrophobic | A/P | A: nonpolar, neutral, hydrophobic; P: nonpolar, neutral, hydrophobic |
|  |  | 232 | Y | polar, neutral | W | nonpolar, neutral |
|  |  | 300 | L | nonpolar, neutral | C | nonpolar, neutral |
|  |  | 510 | W | nonpolar, neutral, hydrophobic | M | nonpolar, neutral, hydrophobic |
